# Malignant perivascular epithelioid cell tumor of mesentery with lymph node involvement: a case report and review of literature

**DOI:** 10.1186/1746-1596-8-60

**Published:** 2013-04-15

**Authors:** Xinge Fu, Ju-hong Jiang, Xia Gu, Zhi Li

**Affiliations:** 1 Department of Pathology, The First Affiliated Hospital, Guangzhou Medical University, 151, Yanjiang Road, Guangzhou, 510120, China; 2 Department of Pathology, The First Affiliated Hospital, Sun Yat-sen University, 58, Zhongshan Road II, Guangzhou, 510080, China

**Keywords:** Perivascular epithelioid cell tumor, Malignant PEComa, Mesentery, Lymph node involvement, Differential diagnosis

## Abstract

**Virtual Slides:**

The virtual slide(s) for this article can be found here: http://www.diagnosticpathology.diagnomx.eu/vs/1309992178882788

Perivascular epithelioid cell tumor (PEComa) is a rare but distinct mesenchymal neoplasm composed of histologically and immunohistochemically unique perivascular epithelioid cells. Due to its relative rarity, little is known about the histogenesis and prognostic factors of this tumor. We describe a case of unusual mesenteric PEComa in a 38-year-old female patient with regional lymph node involvement. Histologically, the tumor was composed of sheet of epithelioid cells with abundant clear or eosinophillic cytoplasms. Extensive coagulative necrosis and a few mitotic figures (2/50 high power field) could be found in tumor. The epithelioid tumor cells were diffusely positive for HMB-45, Melan-A, and focally positive for calponin. One of enlarged mesenteric lymph nodes was observed to be involved by tumor. A diagnosis of malignant mesenteric PEComa with lymph node involvement was made. The patient received chemotherapy after total resection of tumor and segmental resection of involved jejunum. There was no sign of recurrence of tumor found in period of 6-month regular follow-up after chemotherapy. To our knowledge, this is the first case of malignant PEComa in mesentery accompanied with regional lymph node involvement. The literature on this rare tumor is reviewed and diagnostic criteria of malignant PEComa are discussed.

## Background

Perivascular epithelioid cell tumor (PEComa) was first introduced by Zamboni in 1996 to identify a group of mesenchymal neoplasms originating from perivascular epithelioid cells [1]. PEComa family of tumors includes angiomyolipomas (AML), lymphangioleiomyomatosis (LAM), clear cell “sugar” tumor (CCST) of the lung, clear cell myomelanocytic tumor (CCMMT) of the falciform ligament/ligamentum teres and abdominopelvic sarcoma of perivascular epithelioid cells [2]. PEComas other than AML, CCST or LAM are rare. Although increasingly reported over the past decade, PEComas occurring in mesentery are exceedingly rare, with only 6 cases described in the literature so far [3-5]. The clinical behavior of PEComa is not predictable, and there are no strict histologic criteria for malignancy. Because of its relative rarity, little is known about the natural history and prognostic factors of PEComas, although a stratification of PEComa of soft tissue and genitourinary tract has been recently proposed [3,6]. Herein, we report a PEComa of mesentery of small bowel occurring in a middle-aged female patient with mesenteric lymph node involvement. Although the cytologic appearance of the tumor cells was relatively bland, the presence of extensive necrosis and surrounding tissue invasion were indicative of malignant behavior. To the best of our knowledge, this is the first case of malignant mesentery PEComa with lymph node involvement. The literature on this rare tumor is reviewed and differential diagnosis is discussed.

## Case presentation

### Clinical presentation and management

**Figure 1:**
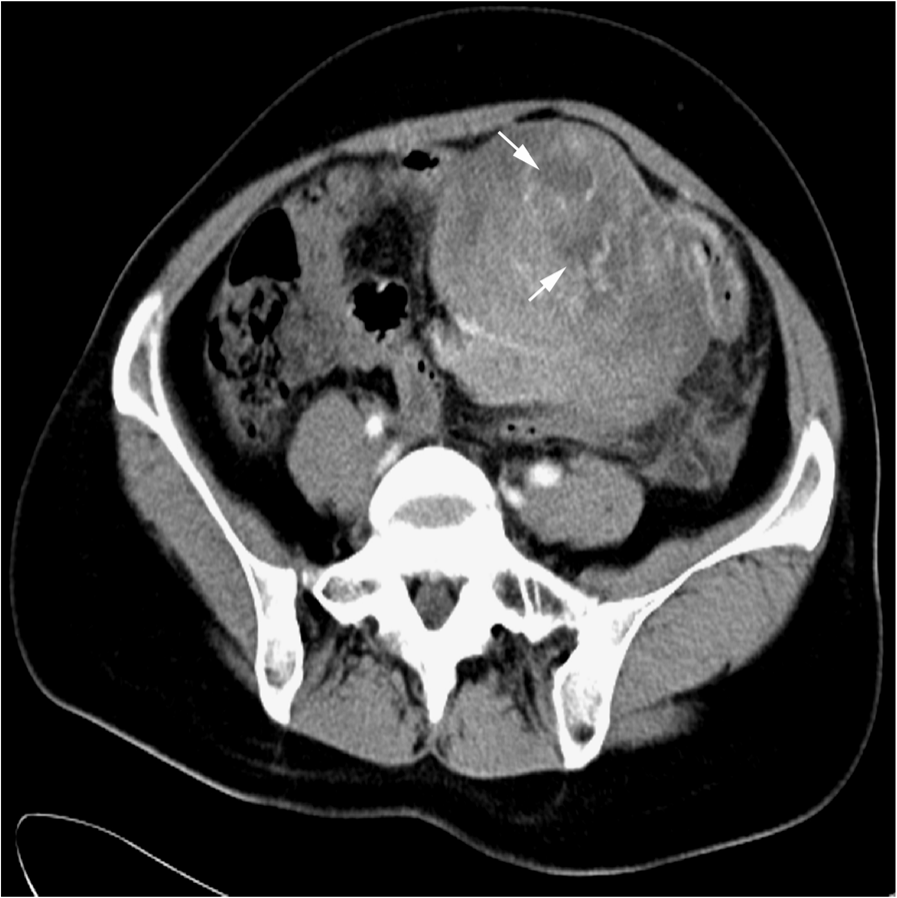
**Preoperative computed tomography (CT) scan of the mesenteric mass.** Contrast-enhanced CT demonstrating a poorly circumscribed solid mass with mild heterogeneous enhancement located at mesentery and showed adhesions of wall of jejunum. The multiple irregular hypointensity areas were observed in the mass and considered to be necrotic areas (white arrow).

A 38-year-old female patient presented with complaints of abdominal pain and abdominal distension that had persisted 3 days before admission to our hospital. Physical examination showed local tenderness and rebounding pain in the left abdominal region, and decreased bowel sounds. The laboratory results, including blood count, differential, liver and renal function, were within the normal range. There was no fever, weight loss and no palpable lymphadenopathy or organomegaly. A computed tomography (CT) scan showed a poorly circumscribed solid mass (9.9 cm × 8.6 cm × 7.1 cm) with mild heterogeneous enhancement located in the left upper abdominal region with adhesions of wall of jejunum. There was hypointensity areas observed in the mass after gadolinium injection (Figure 1). A preoperative presumed diagnosis was extra-gastrointestinal stromal tumor of abdomen. The patient underwent tumor resection and segmental resection of the jejunum. At surgery, the mass was located at the mesentery of the small bowel and a part of the mass was observed to extend to the wall of jejunum. The mass was removed totally, and the postoperative phase was uneventful. Since there was a possibility of tumor metastasis to another anatomical location, the patient was referred to a whole body positron emission tomography (PET)/CT study to search for the potentially secondary tumor, but no abnormality was found. After diagnosis, the patient received 2 courses of chemotherapy with vincristine, ifosfamide, and adriamicin. The patient was on regular follow-up for 6 months after chemotherapy, there was no sign of recurrence of tumor found in this period.

## Material and methods

**Figure 2:**
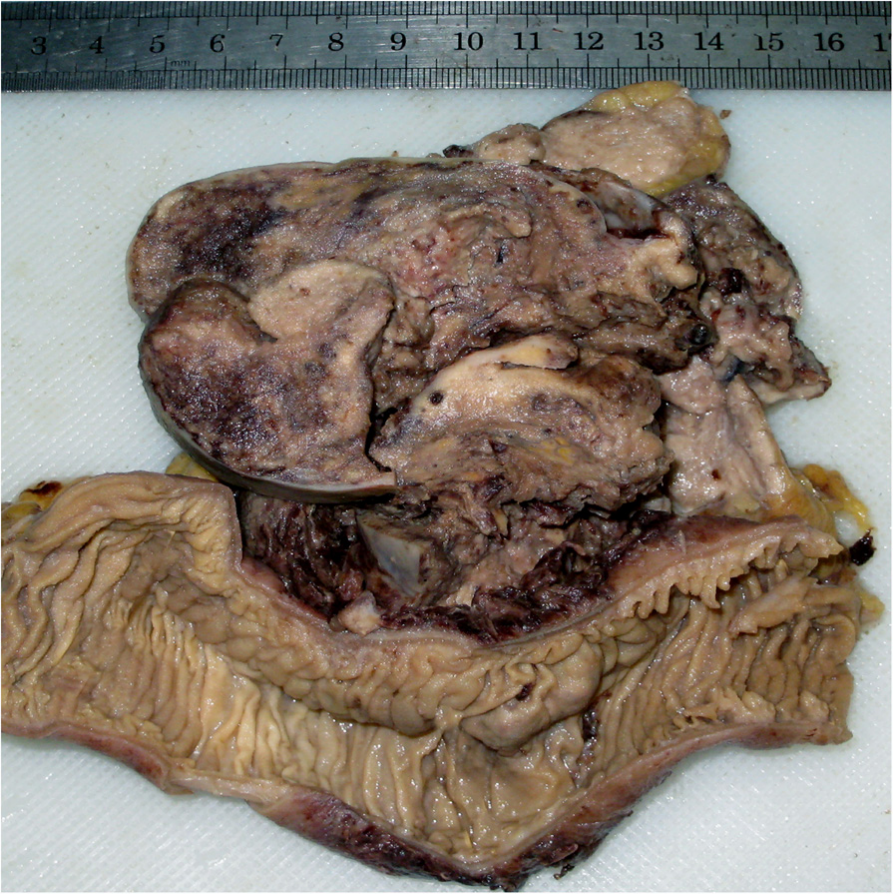
**Gross examination of resected mesenteric mass.** The mass was gray-tan solitary nodular mass without a fibrous capsule. The mass was observed to extend to the wall of jejunum and necrotic areas were also found.

On macroscopical examination, the lesion was gray-tan solitary nodular mass with necrotic areas, measuring 10.0 × 8.5 cm, was located in the mesentery of small intestine. The mass was poorly circumscribed and there was no fibrous capsule round the mass. A part of tumor was observed to extend to the wall of jejunum and some enlarged mesenteric lymph nodes were also observed (Figure 2). The tumor was routinely fixed in 10% neutral buffered formalin and the tissues were embedded in paraffin. Four micrometer-thick sections were stained with Hematoxylin and Eosin (HE). Immunohistochemical analyses were performed using the ChemMate Envision/HRP Kit (Dako, Glostrup, Denmark). The antibodies used in this study were cytokeratin (AE1/AE3), epithelial membrane antigen (EMA), vimentin, S-100 protein, HMB-45, Melan-A,neuron-specific enolase (NSE), synaptophysin (Syn), chromogranin A (CgA), CD34, CD117, Dog-1, smooth muscle actin (SMA), desmin, Myo-D1, CD68, CD99 and Ki-67.

### Pathological findings

**Figure 3:**
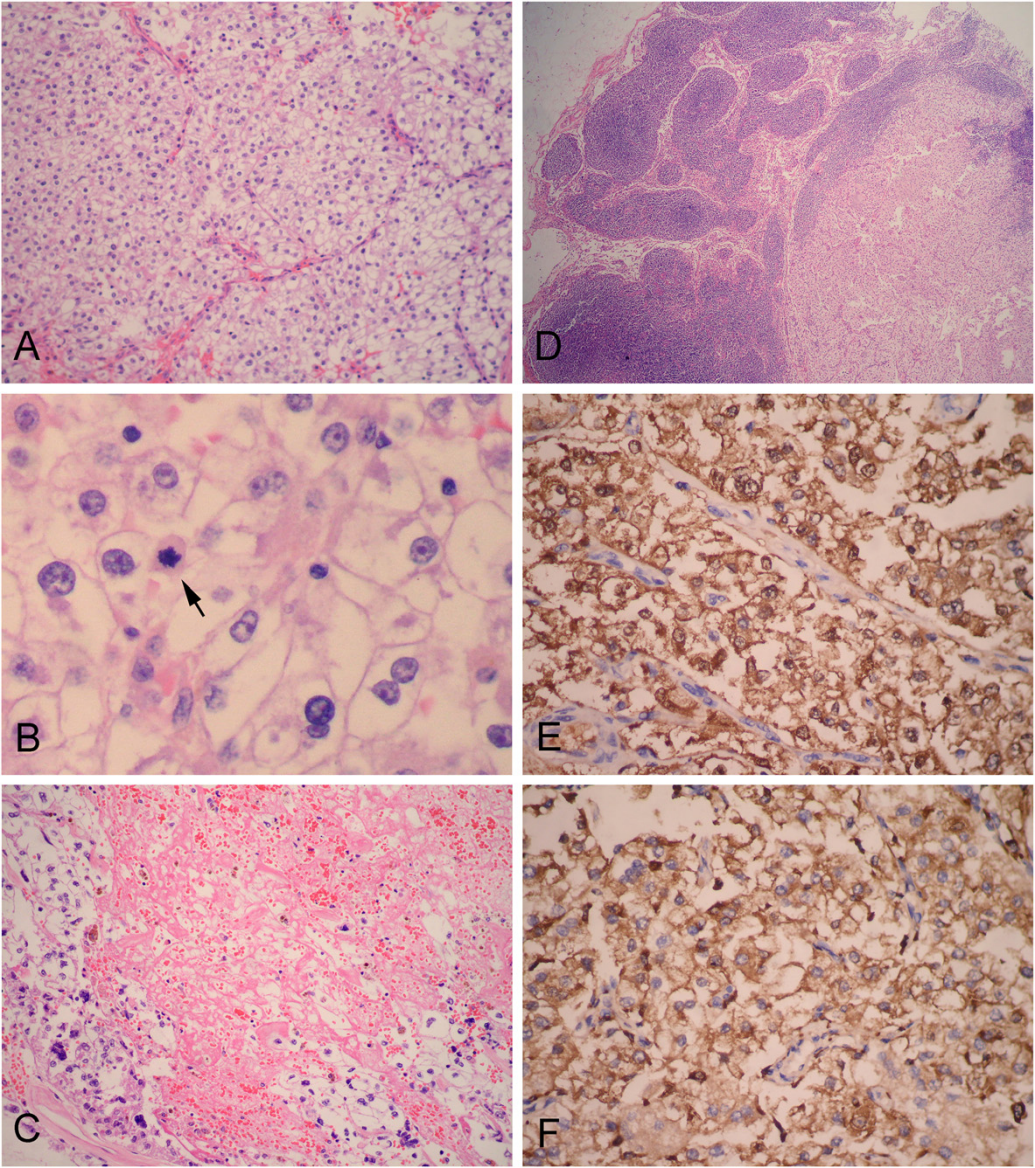
**Photomicrographs of the mesenteric mass.** (**A**) At lower power field, the tumor was observed to be composed of epithelioid tumor cells with clear to eosinophilic cytoplasms. The epithelioid tumor cells were arranged around delicate vasculature. (**B**) The epithelioid tumor cells had round to oval vesicular nuclei with 1-2 centrally located small nucleoli, and mitotic figure was observed in the tumor cells (black arrow). (**C**) Extensive necrotic areas were observed in the tumor. Note the atypical tumor cells with irregular hyperchromatic nuclei at the periphery of necrosis. (**D**) One of enlarged mesenteric lymph nodes was observed to be involved by tumor cells. Immunohistochemical analysis of the mesenteric mass showed epithelioid tumor cells were diffusely positive for melanocytic markers, HMB-45 (**E**) and Melan-A (**F**). (**A** and **C**, H&E staining with original magnification × 200; **B**, H&E staining with original magnification × 400; **D**, H&E staining with original magnification × 100; **E**-**F**, Immunohistochemical staining with original magnification, ×400).

Under microscopic examination, a part of the tumor was surrounded by a thin fibrous pseudocapsule at the periphery. The tumor was composed of cells with abundant clear to fine eosinophilic granular cytoplasm and round, uniform, nuclei arranged in nests or wide fascicles with delicate vascular septa. Most of tumor cells appeared to have bland cytologic features. However, in some areas, the cells became more epithelioid, and nuclei varied in size and shape, with dispersed chromatin and prominent nucleoli. Mitoses were present with mitotic rate of 2/50 high power fields. There was extensive coagulative necrosis and hemorrhage but neither adipocytes nor thick-walled blood vessels were observed in the tumor. The tumor cells appeared to have infiltrated into the underlying smooth muscle of jejunum but there were no features of vascular invasion. One of enlarged lymph nodes of mesentery was found to be involved by tumor (Figure 3A-D). Immunohistochemically, the tumor cells were stained positive for HMB-45, Melan-A, NSE, CD68 and calponin (focal and weakly), but were negative for S-100 protein, Syn, CgA, cytokeratin (AE1/AE3), EMA, CD34, CD117, Dog-1, SMA, desmin, Myo-D1 and CD99. Ki-67 was positive in 3% of tumor cells (Figure 3E-F). Based on the pathological findings, the mesenteric mass was diagnosed as malignant perivascular epithelioid cell tumor with lymph node involvement.

## Conclusions

Perivascular epithelioid cell tumor (PEComa) is a mesenchymal tumor composed chiefly of perivascular epithelioid cell (PEC). PEC was first described in 1994 by Bonetti et al. to introduce the concept of a family of tumor that was characterized by the presence of a peculiar muscle cell that expressed melanoma-associated antigens such as HMB-45 and HMSA-1 [7]. The term PEComa was coined by Zamboni et al. in 1996 to describe this rare family of lesions [1]. In 2002, the World Health Organization accepted the designation PEComa as a distinct mesenchymal neoplasm composed of histologically and immunohistochemically unique PECs [8]. The histogenesis and cytogenetics of PEComa are largely unknown. Until recently, there has been no known normal counterpart of PEC. Bonnetti et al. hypothesized that PEC can modulate its morphology and immunophenotype. They believe that the PEComa is composed of PECs in different stages of modulation with consistent reactivity for melanoma-associated markers, variable reactivity for muscular markers, and nonreactivity for epithelial markers [7]. Although a few researchers still doubt the existence of this entity due to the overlapping morphologic and immunologic features with smooth muscle tumors and the absence of a normal counterpart for PEC, the increasing genetic evidence has shown PEComa to be a distinct type of neoplasm [9,10].

PEComas have been found in various organs and have a tendency to affect women with approximately 40% of tumors originated from the uterus [2], although some unusual sites can be also involved occasionally, such as vagina [11], orbit [12], small and large bowel [13], heart [14] prostate [15], bladder [16] and endometrium [17]. Multifocal PEComas (PEComatosis) have been described in the literature [18]. Mesenteric PEComas are rare with only 6 cases described in the literature so far [3-5] (Table 1). The ratio of mesenteric PEComa incidence in women and men is 2:1. 4 of 6 cases were considered to be malignant PEComa with tissue invasion and the tumors recurred within 6-22 months, although two cases received concurrent chemoradiotherapy after surgical resection. None of previously reported mesenteric PEComas has lymph node involvement. To our best knowledge, our presenting case is the first one of mesenteric malignant PEComa with lymph node involvement, although the cytologic appearance of tumor cells is relatively bland.

**Table 1:** Clinicopathological features of mesenteric PEComas described in present and previous reports

**No.**	**Authors (yr.)**	**Diagnosis**	**Age (year)/Gender**	**Tumor size (cm)**	**Nuclear grade**	**Cellularity**	**MF (per 50HPF)**	**Invasion**	**Necrosis**	**LN status**	**Treatment**	**Outcome**
1	Folpe AL (2005) [3]	PEComa with UMP	67/Female	13.0	High	Moderate	0	No	No	Not involved	SE only	NED at 84 months
2		Benign PEComa	97/Female	4.0	Intermediate	Moderate	0	No	No	Not involved	SE only	NED at 38 months
3		Malignant PEComa	80/Female	9.5	High	High	>50	Vascular invasion	Yes	Not involved	SE only	NED at 19 months
4		Malignant PEComa	46/Female	12.0	Intermediate	Moderate	5	Vascular invasion	Yes	Not involved	SE + CT	Recur and liver metastases at 22 months; die at 27 months
5	Gross E (2010) [4]	Malignant PEComa	5.5/Male	5.0	High	Moderate	NA	Surrounding tissue invasion	No	Not involved	SE only	NED at 24 months
6	Lai CL (2012) [5]	Malignant PEComa	59/Male	11.0	High	High	3	Vascular invasion	Yes	Not involved	SE + CT	Recur at 6 months; alive
7	The present case	Malignant PEComa	38/Female	10.0	Intermediate	Moderate	2	Surrounding tissue invasion	Yes	Involved	SE + CT	NED at 6 months

UMP, uncertain malignant potential; MF, mitotic figure; LN, lymph node; SE, surgical excision; CT, chemotherapy; NA, not available; NED, no evidence of disease.

Due to their rarity, the criteria for the diagnosis of malignancy have not yet been fully established. Some types of PEComa family, such as AML and epithelioid AML of kidney, have been infrequently reported to have metastasis to lymph nodes or extra-renal sites, but regional lymph node involvement and vascular invasion were considered to represent a multifocal growth pattern rather than metastasis [19]. Among adverse pathologic parameters in AMLs, including cellularity, high nuclear grade and mitotic figures, none correlate with outcome, although tumors with necrosis, mitotic activity, nuclear anaplasia and extra-renal spread should raise significant concern for malignant outcome. In fact, we are not sure the lymph node involvement of the current case representing a multifocal growth pattern or metastasis so far. Folpe et al. proposed provisional criteria for PEComas of soft tissue and gynecoelogic origin in 2005 [3]. In these diagnostic criteria, PEComas were classified into “benign,” “of uncertain malignant potential,” and “malignant” categories. There were 6 histological features indicative of high risk: tumor size >5 cm, infiltrative pattern, high nuclear grade and cellularity, high mitotic rate (>1/50 HPF), necrosis, and vascular invasion. Small PEComas (<5 cm) without any of the 6 high-risk features were most likely to be benign. Large PEComas (>5 cm) without any other features had uncertain malignant potential. PEComas with 2 or more high-risk features should be considered malignant. In 2008, Fadare et al. suggested that the only features that indicate a definite potential for aggressive behavior were a mitotic count >1/10 HPF and/or coagulative necrosis, while cytologic atypia should be considered to be at least an indication of uncertain malignant potential. The size of tumors was not advised to distinguish malignancy or non-malignant PEComas [6]. The regional lymph node involvement was not considered to be a worrisome histologic feature in both criteria. The present case was considered to be a malignant PEComa, because the tumor had the most of worrisome histologic features presented either by Folpe or by Fadare. However, it has revealed that malignant PEComa with frankly unfavorable morphological features have an indolent clinical course even in the presence of lymph node involvement [20]. Recent studies have also demonstrated that random X chromosome inactivation could be detected in various involved lymph nodes, supporting the hypothesis of a multifocal disease rather than a metastatic tendency [20]. Due to the limited case numbers and the short follow-up period, we could not define prognostic factors for malignant PEComa. However, combined with previously reported PEComas with malignant features [4,5,15,20,21], we consider that malignant PEComas should be highly aggressive in biological behavior. Therefore, infiltrating growth pattern and extensive coagulative necrosis should be more important factors to be used for malignant evaluation because they represent the aggressiveness in biological behavior of this tumor. Hypercellularity, high or atypical mitotic figures, and even regional lymph node involvement might not indicate the overt malignancy of this tumor.

The microscopic morphology of the present case was characterized by epithelioid tumor cells with abundant clear cytoplasm arranged in nests or wide fascicles with delicate vascular septa. These morphological appearances may pose a diagnostic dilemma, especially in unusual locations. A differential diagnosis of PEComa in soft tissue usually includes clear-cell sarcoma of soft tissue (also known as “malignant melanoma of soft parts”), alveolar soft part sarcoma and epithelioid leiomyosarcoma. However, PEComas differential diagnosis in the mesentery should mainly include epithelioid leiomyosarcoma, epithelioid extra-gastrointestinal stromal tumor (extra-GIST) and metastatic clear-cell renal cell carcinoma. Although PEComas and clear-cell sarcoma of soft tissue share some phenotypic features, including positive reactivity to the melanocytic marker HMB-45 and Melan-A, clear-cell sarcoma of soft tissue exhibits dense fibrous septae rather than the delicate vascular-rich stroma of the PEComas. In addition, clear-cell sarcoma of soft tissue always shows S-100 protein positivity, which is usually absent in PEComa. Alveolar soft part sarcoma is a rare tumor composed of large, uniform, epithelioid cells having abundant eosinophilic, granular cytoplasm arranged in solid nests and/or alveolar structures, separated by thin, sinusoidal vessels, which may cause diagnostic confusion with PEComas. However, alveolar soft part sarcoma has no consistently immuno-positive findings, particularly it is negative for melanocytic markers. Epithelioid leiomyosarcoma may share histologic features of PEComas: both tumors are composed of spindle and/or epithelioid cells with variable reactivity for smooth muscle markers, such as SMA, calponin and H-caldesmon. However, leiomyosarcomas show negative staining for melanocytic markers. In addition, PEComa is readily differentiated from epithelioid extra-GIST and metastatic clear-cell renal cell carcinoma by positive immunohistochemical staining for Melan-A and HMB-45 and negative staining for both CD117, Dog-1, CD34 and pan-cytokeratin (AE1/AE3).

Effective therapies for PEComas have yet to be established and the management of PEComas is quite variable. Surgical excision is the most common approach for PEComa but adjuvant chemo- or radio-therapy, even hormone therapy has also been advised for all patients with malignant features. However, due to the rarity of the disease and the difficulty in predicting the malignant behavior, the benefit of those adjuvant therapies is still questionable. In our case, the patient received 2 courses of chemotherapy after surgery, and there was no sign of recurrence of tumor found in short follow-up period. However, close clinical surveillance accompanied by imaging examination should be recommended to inspect the local recurrence and distant metastasis of this tumor.

In conclusion, we reported herein an unusual case of mesenteric PEComa occurring in a middle-aged patient with regional lymph node involvement. Although the cytologic appearance of tumor cells was relatively bland, the infiltrating growth pattern and extensive coagulative necrosis should be indicative malignancy. We consider that marked hypercellularity, active mitotic figures, and even regional lymph node involvement observed merely in tumor might not represent a distinctive sign of malignant PEComa. However, this postulate should be further investigated by more such cases with long-term follow up.

## Consent

Written informed consent was obtained from the patient for publication of this case report and any accompanying images. A copy of the written consent is available for review by the Editor-in-Chief of this journal.

## Abbreviations

PEComa: Perivascular epithelioid cell tumor; PEC: Perivascular epithelioid cell.

## Competing interests

The authors declare that they have no competing interests.

## Authors’ contributions

XF and JHJ made contributions to acquisition of clinical data, and analysis of the histological and immunohistochemical features. ZL drafted the manuscript. XG revised manuscript critically for important intellectual content and had given final approval of the version to be published. All authors read and approved the final manuscript.

## References

[B1] Zamboni G Pea M Martignoni G Zancanaro C Faccioli G Gilioli E Pederzoli P Bonetti F Clear cell “sugar” tumor of the pancreas. A novel member of the family of lesions characterized by the presence of perivascular epithelioid cells Am J Surg Pathol 1996 20 722 730 10.1097/00000478-199606000-00010 8651352

[B2] Weiss SW Goldblum JR Weiss SW, Goldblum JR Perivascular epithelioid cell family of tumors Enzinger and Weiss’s soft tissue tumors 2008 5 Philadelphia, PA: Mosby Inc 1138 1156

[B3] Folpe AL Mentzel T Lehr HA Fisher C Balzer BL Weiss SW Perivascular epithelioid cell neoplasms of soft tissue and gynecologic origin: a clinicopathologic study of 26 cases and review of the literature Am J Surg Pathol 2005 29 1558 1575 10.1097/01.pas.0000173232.22117.37 16327428

[B4] Gross E Vernea F Weintraub M Koplewitz BZ Perivascular epithelioid cell tumor of the ascending colon mesentery in a child: case report and review of the literature J Pediatr Surg 2010 45 830 833 10.1016/j.jpedsurg.2010.01.015 20385296

[B5] Lai CL Hsu KF Yu JC Chen CJ Hsieh CB Chan DC Li HS Hsu HM Malignant perivascular epithelioid cell tumor of the mesentery: a case report and literature review Onkologie 2012 35 114 117 10.1159/000336826 22414975

[B6] Fadare O Perivascular epithelioid cell tumor (PEComa) of the uterus: an outcome-based clinicopathologic analysis of 41 reported cases Adv Anat Pathol 2008 15 63 75 10.1097/PAP.0b013e31816613b0 18418088

[B7] Bonetti F Pea M Martignoni G Doglioni C Zamboni G Capelli P Rimondi P Andrion A Clear cell (“sugar”) tumor of the lung is a lesion strictly related to angiomyolipoma–the concept of a family of lesions characterized by the presence of the perivascular epithelioid cells (PEC) Pathology 1994 26 230 236 10.1080/00313029400169561 7991275

[B8] Folpe AL Fletcher CDM, Unni KK, Mertens F Neoplasms with perivascular epithelioid cell differentiation (PEComa) World Health Organization Classification of Tumors. Pathology and genetics of tumors of soft tissue and bone 2002 Lyon: IARC Press 221 222

[B9] Pan CC Jong YJ Chai CY Huang SH Chen YJ Comparative genomic hybridization study of perivascular epithelioid cell tumor: molecular genetic evidence of perivascular epithelioid cell tumor as a distinctive neoplasm Hum Pathol 2006 37 606 612 10.1016/j.humpath.2006.01.008 16647959

[B10] Pan CC Chung MY Ng KF Liu CY Wang JS Chai CY Huang SH Chen PC Ho DM Constant allelic alteration on chromosome 16p (TSC2 gene) in perivascular epithelioid cell tumour (PEComa): genetic evidence for the relationship of PEComa with angiomyolipoma J Pathol 2008 214 387 393 10.1002/path.2289 18085521

[B11] Ong LY Hwang WS Wong A Chan MY Chui CH Perivascular epithelioid cell tumour of the vagina in an 8 year old girl J Pediatr Surg 2007 42 564 566 10.1016/j.jpedsurg.2006.10.050 17336201

[B12] Guthoff R Guthoff T Mueller-Hermelink HK Sold-Darseff J Geissinger E Perivascular epithelioid cell tumor of the orbit Arch Ophthalmol 2008 126 1009 1011 18625958 10.1001/archopht.126.7.1009

[B13] Shi HY Wei LX Sun L Guo AT Clinicopathologic analysis of 4 perivascular epithelioid cell tumors (PEComas) of the gastrointestinal tract Int J Surg Pathol 2010 18 243 247 10.1177/1066896908330481 19124450

[B14] Tai Y Wei L Shi H Perivascular epithelioid cell tumor of the heart in a child Pediatr Dev Pathol 2010 13 412 414 10.2350/09-10-0726-CR.1 20085497

[B15] Pan CC Yang AH Chiang H Malignant perivascular epithelioid cell tumor involving the prostate Arch Pathol Lab Med 2003 127 E96 E98 12562263 10.5858/2003-127-e96-MPECTI

[B16] Yin L Bu H Chen M Yu J Zhuang H Chen J Zhang H Perivascular epithelioid cell neoplasm of the urinary bladder in an adolescent: a case report and review of the literature Diagn Pathol 2012 7 183 10.1186/1746-1596-7-183 23276164 PMC3542191

[B17] Fang CL Lin YH Chen WY Microscopic endometrial perivascular epithelioid cell nodules: a case report with the earliest presentation of a uterine perivascular epithelioid cell tumor Diagn Pathol 2012 7 117 10.1186/1746-1596-7-117 22937790 PMC3487803

[B18] Yang W Li G Wei-qiang Z Multifocal PEComa (PEComatosis) of the female genital tract and pelvis: a case report and review of the literature Diagn Pathol 2012 7 23 10.1186/1746-1596-7-23 22404894 PMC3378439

[B19] Abdulla M Bui HX del Rosario AD Wolf BC Ross JS Renal angiomyolipoma. DNA content and immunohistochemical study of classic and multicentric variants Arch Pathol Lab Med 1994 118 735 739 8024411

[B20] Alaggio R Cecchetto G Martignoni G Bisogno G Cheng L Sperlì D d'Amore ES Dall'Igna P Malignant perivascular epithelioid cell tumor in children: description of a case and review of the literature J Pediatr Surg 2012 47 e31 40.l 22703822 10.1016/j.jpedsurg.2012.02.023

[B21] Armah HB Parwani AV Malignant perivascular epithelioid cell tumor (PEComa) of the uterus with late renal and pulmonary metastases: a case report with review of the literature Diagn Pathol 2007 2 45 10.1186/1746-1596-2-45 18053181 PMC2213634

